# Dr. Joseph S. Hartman

**Published:** 1881-12

**Authors:** 


JOSEPH S. HARTMAN, D. D. S , died of consumption, at Richmond, Va., November 6th, 1881, in the twenty-fifth year of his age. Dr. Hartman was a graduate of the Baltimore College of Dental Surgery of the class of 1879, and soon after graduating became associated in the practice of his profession with Dr. Geo. B. Steele, of Richmond, with whom he remained up to the time of his death.

He was a young man of great promise, and had his life been spared would no doubt have become a prominent dental practitioner. His manners were quiet and unassuming, but characterized by energy and close application, and his character was above reproach.

